# Patterns of understory invasion in invasive timber stands of a tropical sky island

**DOI:** 10.1002/ece3.9995

**Published:** 2023-04-13

**Authors:** Varughese Jobin, Arundhati Das, C. P. Harikrishnan, Ritobroto Chanda, Swapna Lawrence, V. V. Robin

**Affiliations:** ^1^ Indian Institute of Science Education and Research (IISER) Tirupati Karakambadi Road Tirupati India; ^2^ National Centre for Biological Sciences Bengaluru India; ^3^ Present address: Centre for Ecological Sciences Indian Institute of Science CV Raman Road Bengaluru 560012 India; ^4^ Present address: Wildlife Institute of India Wildlife Institute Rd, Chandrabani Dehradun Uttarakhand 248001 India

**Keywords:** *Eucalyptus*, forest–grassland management, grassland restoration, lantana, secondary invasion, shola Sky Islands

## Abstract

Current climate and land cover change threaten global mountaintops with increased spread of invasive species. Long‐established plantations of invasive trees on these mountaintops can alter their surroundings, further increasing invader‐facilitated invasion. Identifying the ecological conditions promoting such associations can help develop better management interventions. The Western Ghats's Shola Sky Islands (>1400 m MSL) host vast stretches of invasive tree plantations that sustain the colonization of other invasive woody, herbaceous, and fern species in their understories. Here, we analyzed vegetation and landscape variables from 232 systematically placed plots in randomly selected grids using non‐metric multidimensional scaling and Phi coefficient approaches to examine patterns of association (positive interactions) between understory invasive species with specific invasive overstory species. We also conducted GLMM with zero inflation to determine the influence of environmental variables where such associations occur. We find that understory invasion of multiple species under the canopy of other invasives is widespread across the Shola Sky Islands. Stands of *Eucalyptus* host the colonization of 70% of non‐native invasive species surveyed across the Shola Sky Islands. In particular, the *Lantana camara* invasion is strongly associated with *Eucalyptus* stands. We also found that climatic variables affect the colonization of understory woody invasive species, while invasion by exotic herbaceous species is associated with the density of road networks. Canopy cover impacts all invasives negatively, while fire incidence was negatively associated with invasion by *Lantana* spp. and the *Pteridium* spp. While the restoration of natural habitats primarily targets the highly invasive *Acacia*, less invasive *Eucalyptus* and *Pinus* are often not included. Our study suggests that retaining such invasive species in natural habitats, particularly protected areas, can hinder ongoing grassland restoration efforts by facilitating further invasions by multiple woody and herbaceous species.

## Graphical abstract

Long‐established stands of invasive timber trees on mountaintops with unique natural habitats can increase invader‐facilitated invasion. The study identifies the ecological conditions that promote such specific associations and can help develop better management interventions.

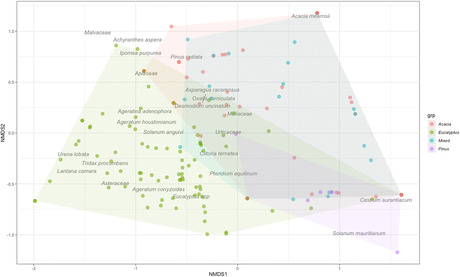

## 1 INTRODUCTION

Invasive plant species have great potential to expand within global biodiversity hotspots (Wan & Wang, 2018) and impact endangered species (Dueñas et al., 2021). Mountaintops face increasing threats from invasive species owing to the ongoing changes in climate and land cover (McDougall et al., 2011; Pauchard et al., 2009). Across several tropical mountaintops or sky islands, planted timber trees (*Acacia*, *Eucalyptus* & *Pinus*) have escaped the originally managed planted sites and invaded a much larger landscape. Such non‐native invasive tree species have established extensive stands (henceforth referred to as “timber stands”) during the past century (Arasumani et al., 2019; Hulme et al., 2013), posing serious threats to native ecosystems (Hejda et al., 2017). Apart from their negative impacts on native regeneration (Dyderski et al., 2017), studies from low‐elevation ecosystems show that timber stands can also display signs of a heterospecific invasion (Kuebbing & Nuñez, 2015; Tecco et al., 2007), particularly in the understory. Studies from mountains on such invasions followed by changes in ecological circumstances by a primary invader are few (Giantomasi et al., 2008) and, thus, warrant further exploration.

Invasive trees introduced in grassland–forest mosaics in high‐altitude mountains (Arasumani et al., 2019; Hulme et al., 2013) impact biodiversity, water, and soil systems with their invasion into grasslands (Simberloff, 2011; van Wilgen & Richardson, 2014). Timber stands can alter their local habitat, and these effects get multiplied due to their size and longevity (Dyderski et al., 2017; Le Maitre et al., 2011). Their presence and influence may also facilitate the invasion of other non‐natives, especially in the understory (Kuebbing & Nuñez, 2015; O'Loughlin & Green, 2018). Such understory invasives can have cascading impacts by competing with natural forest regeneration (Dyderski et al., 2017; Vacek et al., 2020), impacting animal movements (Habel et al., 2016; Stewart et al., 2021), and enhancing edge effects (McDonald & Urban, 2006). Mapping such invasions at a landscape level becomes imperative to understand if particular overstory invasive species are associated with specific understory invasive species. Large‐scale management of such timber stands on mountains can pose a great challenge to forest managers due to complex topography and dense vegetation (McDougall et al., 2011). Identifying stands with significant understory invasion may ease management through targeted interventions (Blackburn et al., 2011; Mack et al., 2000).

The Shola Sky Islands or the mountaintops of the Western Ghats (Robin & Nandini, 2012)—a UNESCO World Heritage site (UNESCO, 2012) and global Biodiversity Hotspot (Myers et al., 2000)—are a good system to study understory invasion in long‐established timber stands. In the Shola Sky Islands, *Acacia*, *Eucalyptus* and *Pinus* trees were planted in the Shola grasslands (considered wastelands) by the East India Company in the 1800s as timber crops to ease the region's firewood scarcity (Joshi et al., 2018). Later, planting and management of these trees through extraction at different ages/stages continued until 1996. By this time, apart from the planting, these trees had already become invasive in parts of the landscape. In 1996, the Supreme Court of India prohibited tree felling (Rosencranz & Lélé, 2008), yet the planting continued. Another court order in 2014 directed the Tamil Nadu State Forest Department to remove invasive trees from the landscape (Correspondent, 2015) and to restore the original, native shola–grassland mosaic habitat. The Forest Department is currently removing some of these invasive and non‐native tree stands in an effort to restore native grasslands. However, timber stands of mixed ages and different species continue to occupy a large proportion of the landscape (40% of the study area; Arasumani et al., 2019; Joshi et al., 2018). Today, the landscape is a complex system of timber stands of three species; *Acacia mearnsii*, one of the world's most invasive species (Global Invasive Species Database, 2010), *Eucalyptus globulus*, and *Pinus radiata* (henceforth, *Acacia*, *Eucalyptus*, and *Pinus*, respectively) all known for their invasibility globally (Richardson & Rejmánek, 2011). They form widespread single‐ or multi‐species stands across the Shola Sky Islands. Several other invasive species are known to colonize the understories of these stands with sporadic observations across the Shola Sky Islands (Balaguru et al., 2016; A. Das Pers. Obs.). Species like *Ageratina* spp., *Ageratum* spp., *Cestrum aurantiacum*, *Lantana camara*, and *Pteridium aquilinum* (henceforth, *Ageratina* complex, *Cestrum*, *Lantana*, and *Pteridium*, respectively) have invaded multiple locations across Shola Sky Islands (Balaguru et al., 2016; Das, 2015). These species are considered invasive globally (Bhatt et al., 2011; Cowie et al., 2018; Goncalves et al., 2014; Makokha, 2018; Marrs & Watt, 2006; Wan et al., 2010). Most interactions between the invasive overstory species and invasive understory species are thought to be neutral or negative (Kuebbing & Nuñez, 2015), but positive interactions are uncommon (Gómez‐Aparicio, 2009). Such associations need to be highlighted to understand species ecology and manage the spread of invasives.

Based on the interactions between the over‐ and understory of the timber stands, the community of colonizing invasives varies between the types of stands (Orians et al., 2012; Wei et al., 2020). Attributes of overstory trees that affect the understory community include canopy cover (Wei et al., 2020), nitrogen‐fixing abilities (Kuebbing & Nuñez, 2015), basal area (Zilliox & Gosselin, 2014), soil moisture (Wei et al., 2015), and allelopathy (Fisher, 1980). Monocultures of *Acacia*, *Pinus*, and *Eucalyptus* with sparser canopies show greater regeneration capacity of heterospecific understory species (Arevalo et al., 2011; Forbes et al., 2016; Gwate et al., 2016). *Acacia*'s dense stands can hinder heliophilic species' regeneration (Gwate et al., 2016). *Acacia* stands can also show extreme allelopathy, impeding heterospecific regeneration (Fatunbi et al., 2009). On the other hand, studies also indicate that these stands improve soil nitrogen dynamics (Forrester et al., 2007), which may improve regeneration conditions. Stands of *Pinus radiata* show fewer allelopathic restrictions on the growth and germination of invasives compared to *Acacia mearnsii* (Souto et al., 2001). *Eucalyptus globulus* stands show little evidence of allelopathy, and the soil in the stand usually promotes germination and seedling growth of other species (Nelson et al., 2021). However, stands with individuals of *Acacia* and *Eucalyptus* trees may have better soil‐based nitrogen than monocultures of *Eucalyptus globulus* (Forrester et al., 2007).

Invasive species show limited niche expansion beyond their native niches in environmental spaces (Liu et al., 2020). In the Shola Sky Islands, the common understory invasive species include *Ageratum conyzoides*, *Ageratum houstonianum*, *Ageratina adenophora*, *Lantana camara*, *Cestrum aurantiacum*, and *Pteridium aquilinum* (Tamil Nadu policy on invasive alien plant species and ecological restoration of habitats—https://www.forests.tn.gov.in/app/webroot/img/document/news/news/TNPIPER_plants‐1.pdf). Several of these thrive in habitats across the globe (Amouzgar et al., 2020; Junaedi, 2013; Lamsal et al., 2019; Ojunga et al., 2020). In novel or modified habitats of timber stands, we expect the understory invasives to thrive in niche spaces similar to their places of origin. Their response to environmental gradients may also be affected due to the differences in life forms of the colonizing species (Barbier et al., 2008; Zilliox & Gosselin, 2014; details in Table S1).

In our study, we identified invasive species colonizing the understory of timber stands and conducted a region‐wide assessment to answer the following questions:

1. Are there associations of invasive understory species with specific invasive overstory species?
2. How do landscape factors and vegetation structure of timber stands affect the pattern of understory invasion?

We expect timber stands that provide similar microclimatic conditions associated with the original habitats of the colonizing invasives to demonstrate colonization. With their sparser canopy cover and milder allelopathic effects, *Eucalyptus* stands may be expected to show higher species richness of invasives. *Acacia* and *Pinus* may show the least species richness. Woody invasives may prefer warmer plots with greater human disturbances, while herbaceous invasives might thrive in open timber stands with higher soil moisture. Ferns may prefer wetter and warmer areas.

## 2 METHODS

**FIGURE 1:**
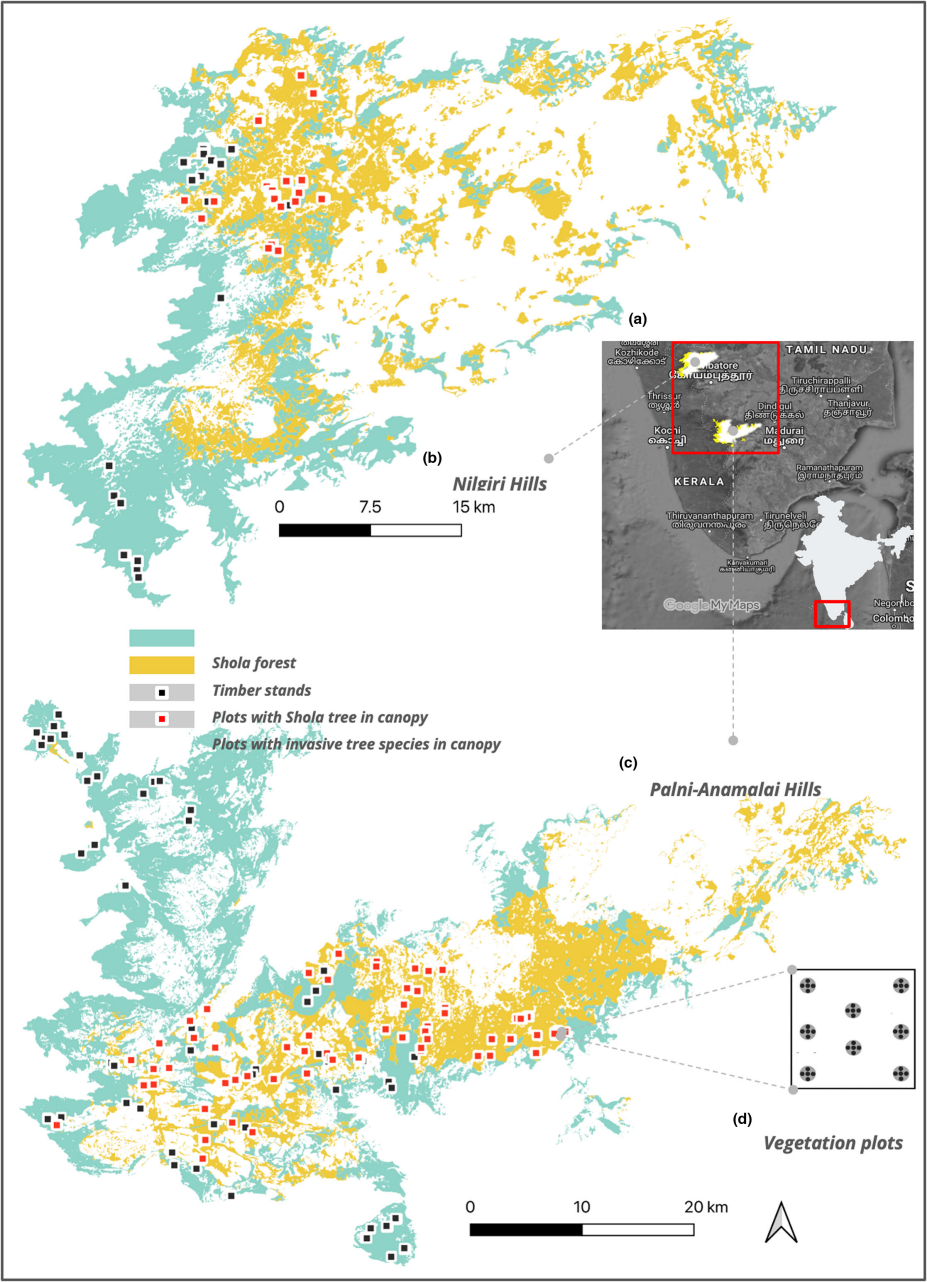
Location of 143 study grids (200 m × 200 m). Inset map (a) shows the location of the Shola Sky Islands of Nilgiri Hills (b) and Palani–Anamalai Hills (c), situated in southern India. We surveyed 596 plots (7 m radius; ~0.015 ha) within these grids, up to eight plots in each grid (d). The red grids have Acacia, Pinus, or Eucalyptus as overstory trees that we sampled with 232 plots for further analysis.

We conducted this study across the high‐elevation montane forests of the Nilgiris and Anamalai–Palani hills landscapes of the Shola Sky Islands in the Western Ghats (10.12°N 77.60°E to 11.50°N 76.70°E; inset A in Figure 1). We classified wooded habitats above 1400 m MSL into Shola forests (Robin & Nandini, 2012) and timber stands, following Arasumani et al. (2019).

Our study sites fall in the Kodaikanal Wildlife Sanctuary, Anamalai Tiger Reserve, Mukurthi National Park in Tamil Nadu, Munnar Wildlife Division, and Silent Valley National Park Kerala (Figure 1). We divided the study regions into 200 m grids and randomly selected 0.5% of those. After omitting inaccessible grids, we had 143 study grids (Figure 1). We laid up to eight plots (7 m radius each) at specific locations within each grid (indicated in inset D in Figure 1). As our study targeted only wooded habitats, we only collected vegetation data from the plots within the grid if they were wooded. If the plot fell within open landcover types (agricultural fields, tea plantations, grasslands, and settlements), we excluded it from the study. Hence, the number of plots within the study grids varied from two to eight. We sampled 143 study grids containing a total of 596 plots across this landscape. Our sampling of wooded habitats included both native Shola forests and non‐native timber stands. However, our study questions were focused only on the understories of non‐native timber stands. So, we excluded a further 364 plots that had native tree species in the canopy. Therefore, our final analysis was based on 232 plots that had only non‐native trees in the canopy (Figure 1).

### 2.1 Vegetation sampling

In the 7‐m‐radius circular plots (inset D in Figure 1), we identified and measured the circumference at breast height (GBH) for all trees over 30 cm (James & Shugart, 1970). For the analyses, we calculated the total basal area of trees of species: *Eucalyptus*, *Acacia*, and *Pinus* within each plot. In each plot, we placed five sub‐plots (four at the edge along each cardinal direction and one at the center). At the center of each sub‐plot, we measured the vegetation profile using a 5 m pole, calibrated at every 0.5 m (Karr, 1971). The vegetation profile was determined by any contact of the vegetation with the pole. The intensity of regeneration may depend on the amount of light reaching the floor, determined by the canopy cover, measured at those five sub‐plots using HabitApp (Bianchi et al., 2017).

We created a relatively exhaustive list of invasive species from literature and field observations; all of these species are described in the State policy on invasives “Tamil Nadu policy on invasive alien plant species and ecological restoration of habitats.” Within the five, we documented several invasive species, of which the following species were the most common: *Ageratum conyzoides*, *Ageratum houstonianum*, *Ageratina adenophora*, Lantana *camara*, *Cestrum* spp., *Solanum* spp., and *Pteridium aquilinum* and the conspecifics, *Acacia mearnsii*, *Eucalyptus globulus*, and *Pinus* spp. Their heights were also noted. Other invasive species mentioned in the policy were also noted, and their heights were measured.

Since fire is known to promote the spread of certain invasives, for example, *Pteridium aquilinum* (Carvalho et al., 2022), and control the spread of others, for example, *Lantana camara* (Hiremath & Sundaram, 2005), we quantified the extent of fire in our plots. At each of the five sub‐plots in the plots (four cardinal directions and one in the center), we noted the presence of recent fire incidents (burnt understory and blackened bases of the tree trunks). If all the sub‐plots in the plot indicated the presence of fire, the plot would get a 100% score, but several plots were only partially burnt.

We summed the number of leaf contacts in all strata within each plot along with the species‐wise basal area and tree count. The canopy cover was averaged within each plot. We also summed the number of regenerations for each invasive species within each plot.

### 2.2 Environmental variables

We selected putative predictors (described further below) for determinants of the intensity of regeneration (i.e., seed germination, seedling establishment, and sapling growth) in timber stands based on available literature (Amouzgar et al., 2020; Dolling, 1999; Junaedi, 2013; Lamsal et al., 2019; Ojunga et al., 2020; Parthasarathy et al., 2011; Prasad, 2012; Silva & Matos, 2006; Sundaram & Hiremath, 2012; Wan et al., 2010; Yuan & Wen, 2018). We assessed the following variables for each plot; the rationale for their inclusion in the analyses is from literature indicated with the variable: elevation (Arévalo et al., 2005), slope (Cerdà & García‐Fayos, 1997), aspect (Winkler et al., 2016), topographic position index (TPI; Frey & Ashton, 2018), terrain ruggedness index (TRI; Rhodes & St. Clair, 2018), topographic wetness index (TWI; Petroselli et al., 2013), and topographic convergence index (TCI; Bunn et al., 2005). We extracted topographic variables using the BHUVAN Cartosat‐1 DEM v.3 from tiles (30 m contour interval) of our study area in QGIS3.10 Geospatial Data Abstraction Library plugin. We collected TPI, TRI, and TWI at the local scale and TCI at scales of 30, 60, and 90 m after resampling the DEM. The aspect in radians was converted into two variables: the sine aspect (“eastness” of the plot) and the cosine aspect (the “northness” of the plot) because southern and western aspects in the Northern hemisphere receive greater solar irradiation (Piedallu & Gégout, 2008; Stage & Salas, 2007).

We calculated the area of Shola forests within a buffer of 5 ha around each plot (ln_shlbfr5ha) using data from Arasumani et al. (2019). Within the same buffers, we extracted the lengths of roads for each plot (rds_lngth_5ha). The data of the road network were procured from OpenStreetMap (https://www.openstreetmap.org) as a shapefile and clipped to the extent of the 5Ha buffers. The length of road networks within buffers may provide an indicator of the intensity of human access/disturbance for each plot (Benítez‐López et al., 2010; Pauchard et al., 2009).

We also collected climatic variables for each plot studied from the CHELSA (Climatologies at high resolution for the earth's land surface areas) data which consist of downscaled model outputs of temperature and precipitation estimates at a horizontal resolution of 30 arcsecs (Karger et al., 2017). We extracted climatic variables influencing germination and plant growth: the temperature seasonality (Wright, 1996), precipitation in the dry quarters (e.g., Howe, 1990; Martínez‐Ramos et al., 2009), precipitation in the cold quarters (Marques & Oliveira, 2008), the minimum temperature in the cold quarter (Joshi et al., 2020), and the maximum temperature in the hot quarters (Wright, 1991).

### 2.3 Analyses

To assess the adequacy of our sampling, we plotted the species–area curve for each stand type.

#### 2.3.1 Ordination to represent the pairwise dissimilarity between sites

We assessed associations of invasive understory species with specific invasive overstory species using non‐metric multidimensional scaling (NMDS) with the Bray–Curtis distance as the dissimilarity measure (Minchin, 1987). Bray–Curtis is good at detecting underlying ecological gradients (Gauch Jr., 1973). We used the package *vegan* (Oksanen et al., 2013) with R (R Core Team, 2013) and calculated the ordination using the function metaMDS. The dimensions were kept at 4, the maximum number of tries was 500, and the maximum number of iterations in the single NMDS run was 999. We visualized our NMDS plots using *ggplot2* (Wickham, 2016). We computed the subset of points on the convex hull created based on the categorical variable—the stand type. To statistically validate the composition affinity to the stand types, we performed the one‐way ANOSIM nonparametric test with function *anosim* using *vegan* (Oksanen et al., 2013). We examined two parameters for statistical significance and the degree to which the ordinations are related—a *p*‐value and an *R*‐value, respectively. *R*‐values between .25 and 1 indicate a considerable difference (Polanía et al., 2020).

#### 2.3.2 Phi coefficient of association

The Phi coefficient of association (Tichy & Chytry, 2006) treats the target unit and the species symmetrically (joint fidelity measure), which implies that an invasive taxon found exclusively in a particular stand type will have higher fidelity values. The strength of association between species assemblages and stand groups may indicate degrees of preferences for specific site groups. We used the function “*multipatt*” of the package *indicspecies* (De Caceres & Jansen, 2016). We computed 95% confidence intervals using 999 permutations with the argument *func* as *r.g*.

#### 2.3.3 Data pre‐treatment

We pooled the species *Ageratum conyzoides*, *Ageratum houstonianum*, and *Ageratina adenophora* (*Ageratina* complex), given very few records of the latter two. Our final species analysis list included the following taxa: *Ageratina* complex, *Cestrum aurantiacum*, *Pteridium aquilinum*, and *Lantana camara*. To evaluate secondary invasion, we did not consider the regeneration of overstorey species—*Eucalyptus*, *Acacia*, and *Pinus* in the understory; these individuals were excluded from the analyses. We could not conduct analyses for *Solanum mauritianum*, *Solanum* sp., *Ipomea* sp., *Clitoria ternatea*, *Tridax procumbens*, *Asparagus racemosum*, *Desmodium uncinatum*, *Oxalis corniculate*, *Urena lobata*, *Achyranthes aspera*, *Asteraceae*, *Urticaceae*, *Meliaceae*, *Malvaceae*, and *Apiaceae* as the number of plots with these species regenerating was less than five. We removed the single plot with a mix of *Eucalyptus*–*Acacia*–*Pinus* canopy from the analysis for *Lantana* alone.

Pearson correlation coefficient (*r*) was calculated among all pairs of variables. Using function *vif* from R package *usdm* (Naimi et al., 2014), we assessed multicollinearity further by calculating variance inflation factors for each invasive taxon (Dormann et al., 2013). The selected variables had pairwise *r* values lower than .7. The variance inflation factors were under 3.0 for the *Ageratina* complex, *Cestrum*, and *Pteridium* and under 7.0 for *Lantana* (Johnston et al., 2018). All chosen variables for all taxa are presented in Table S1.

Spatial autocorrelation for the regeneration data was assessed using the Mantel test with 1000 Monte Carlo permutations using *mantel.rtest* function from package *ade4* (Dray and Siberchicot, 2007). For each taxon, the simulated *p*‐values were between .1 and .9, indicating no correlation between regeneration data (excluding zero regeneration) and the respective Euclidean distance matrices.

To account for the excess of absences of regeneration in the dataset, we fit a generalized linear mixed‐effect model with zero inflation to the data using the glmmTMB package (Magnusson et al., 2020) in R. A plausible situation may be that some species do not regenerate in some regions because of environmental conditions or other factors, ultimately leading to the absence of that species in those regions. On the other hand, in some regions, even with facilitative conditions, the plots with zero regeneration can still occur by chance. The zero‐inflation component models the absences in the former case, while the count component models them in the latter case.

#### 2.3.4 Model fitting

We tested the predictors for regeneration for each taxon in the count component of the model. For the zero‐inflated component of the model, which models the absence of regeneration, we chose TWI (a proxy for soil moisture), road length within a 5‐ha buffer (a proxy for the source of propagules), and canopy cover (a proxy for light intensity).

We rescaled continuous independent variables to zero mean and unit variance using the function *decostand* (vegan package). We used the glmmTMB package to fit the models and function *AIC* from package *stats* to extract each model's Akaike information criterion corrected for small sample sizes (AICc). We fitted models in all possible combinations of the count component variables and the zero‐inflation component variables for each taxon (Monteiro‐Henriques & Fernandes, 2018), using the function *combn* from the *utils* package (Maintainer, 2016).

#### 2.3.5 Variable importance value

We calculated the AIC differences to the best model for each fitted model. We extracted model weights with function *weights* in package *stat*s (R Core Team, 2013). Variable importance values (VIV) were computed at the end for each modeled variable by adding the Akaike weights of models consisting of the variable (Burnham et al., 2011; Monteiro‐Henriques & Fernandes, 2018). We modified the depiction of effect signals consistency with box plots for variable effects of each taxon studied (Monteiro‐Henriques & Fernandes, 2018). The signal of effect was considered consistent when the interquartile range did not overlap zero.

## 3 RESULTS

Of the 596 plots sampled, invasive trees formed the overstory in 232 plots, and these were selected for this study. Different invasive species, individually and in combinations, formed the overstory across this landscape—*Eucalyptus* 95 (41%) plots, *Acacia* 61 (26%), and *Pinus* 28 (12%); *Acacia* + *Eucalyptus* 33 (14%); other 7% of the total plots had other combinations of the three invasive species in the overstory.

### 3.1 Which stands have the greatest number of understory invasives?

We identified 24 species of invasives, at least to their family: *Ageratum conyzoides*, *Ageratum houstonianum*, *Ageratina adenophora*, *Lantana camara*, *Cestrum aurantiacum*, *Solanum mauritianum*, *Solanum* sp., *Pteridium aquilinum*, *Ipomea* sp., *Acacia mearnsii*, *Eucalyptus globulus*, *Pinus* spp., *Clitoria ternatea*, *Tridax procumbens*, *Asparagus racemosum*, *Desmodium uncinatum*, *Oxalis corniculata*, *Urena lobata*, *Achyranthes aspera*, *Asteraceae*, *Urticaceae*, *Meliaceae*, *Malvaceae*, and *Apiaceae*. *Ageratina* complex shows the most frequency (51.5%), followed by *Acacia mearnsii* (17.7%), *Pteridium aquilinum* (11.0%), *Cestrum aurantiacum* (7.6%), *Lantana camara* (7.0%), and *Eucalyptus* spp. (3.2%).

**FIGURE 2:**
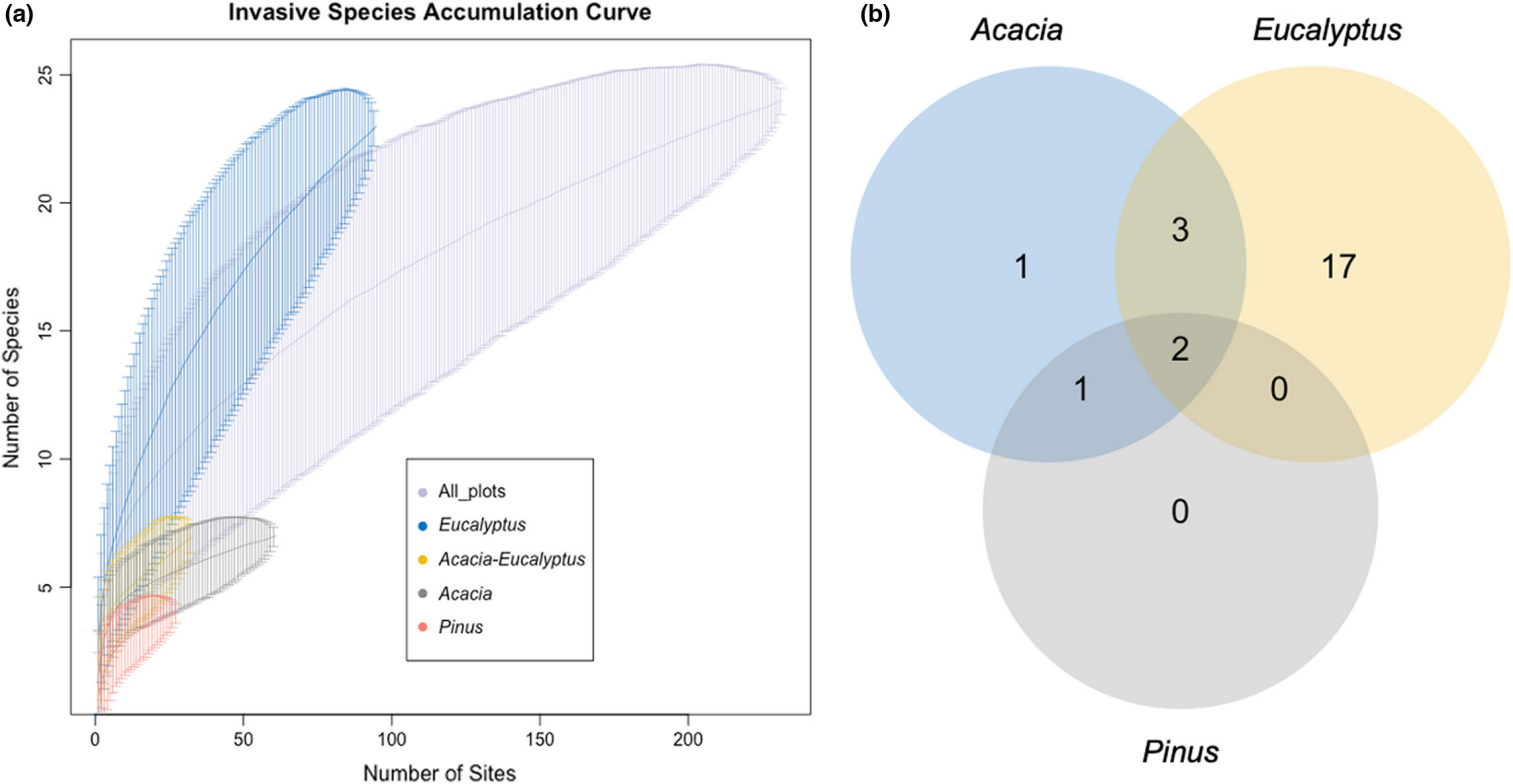
(a) Species accumulation curves for invasive regeneration in each timber stand type, and (b) Venn diagram representing the species richness of understory invasives within each timber stand type.

**FIGURE 3:**
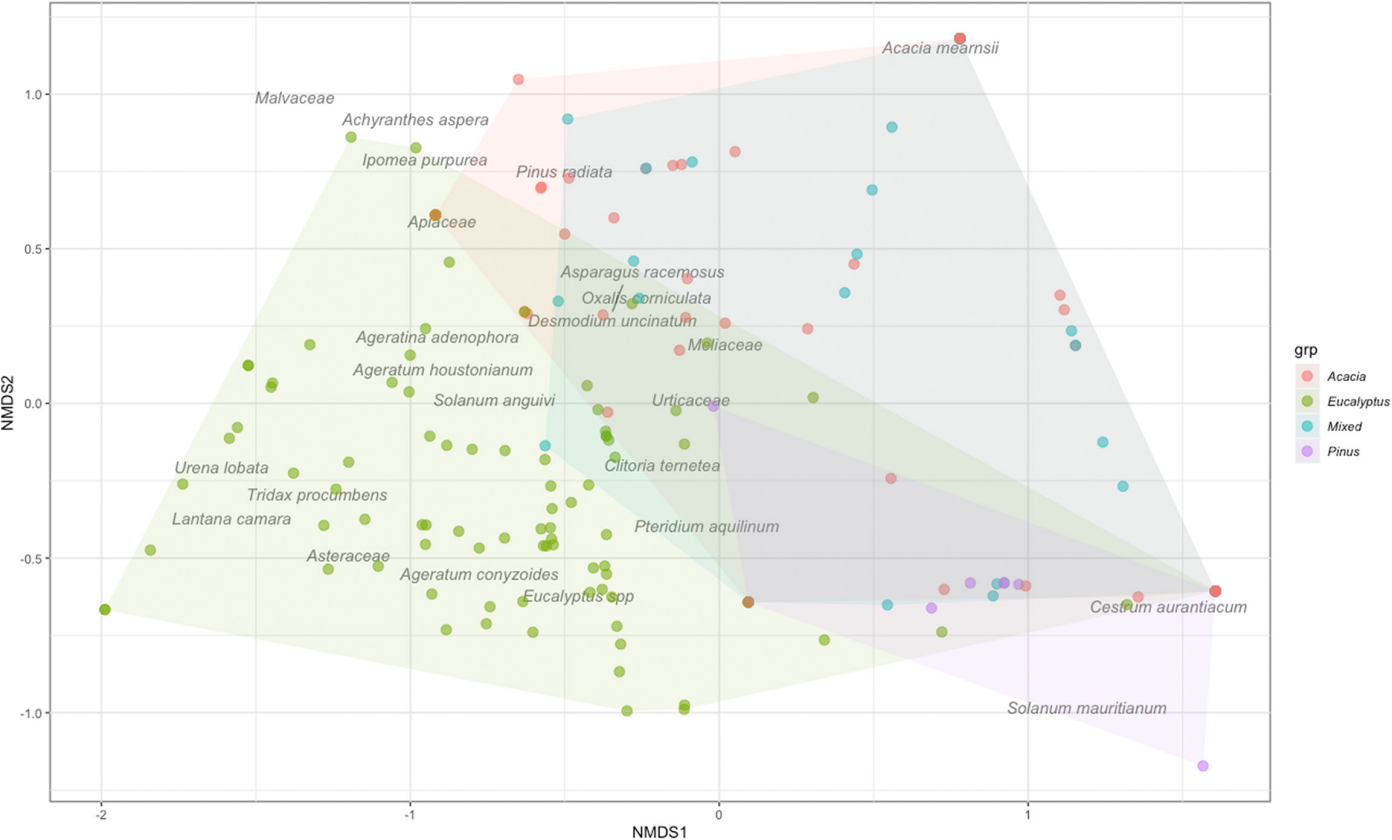
NMDS ordination: Invasive community analyzed by stand type—Eucalyptus stands were different from Acacia and Pinus, which were more similar to each other. The closer points are more similar with respect to the presence and absence of the species within the plots.

*Eucalyptus* stands host the maximum number of regenerating invasive species (17), followed by *Acacia‐Eucalyptus* stands (3) (Figure 2). Monocultural stands of *Pinus* did not have any invasive regeneration in the understory. The final NMDS stress was 0.05500354. Stress <0.1 provides a good representation in reduced dimensions. The sites with different stand overstory types show a significant difference (ANOSIM; *p* < .001, *R* = .32) with regard to the invasive taxon regenerating in the understory (Figure 3).

The species–area curves were steep for stands of *Eucalyptus* and all stands considered cumulatively, but not for *Acacia* and *Pinus* stands. This indicates that more invasive species may be hosted in the understory of invasive trees than we have uncovered with a sampling of 232 plots.

### 3.2 Are there specific associations between overstory species and understory invasives of timber stands?

Species with fidelity values above 0.4 are usually diagnostic for the target vegetation unit (stand types: *Acacia*, *Eucalyptus*, *Pinus*, and Mixed). *Lantana* was the sole taxon that showed high fidelity to *Eucalyptus* stands (fidelity value = 0.429, *p* = .002; Table S2).

### 3.3 Relationship of understory invasives with habitat and environmental variables

Variable importance values based on Akaike weights identify the most relevant variables for the distribution of each invasive taxon in both count and zero‐inflation components (Table S3).

**FIGURE 4:**
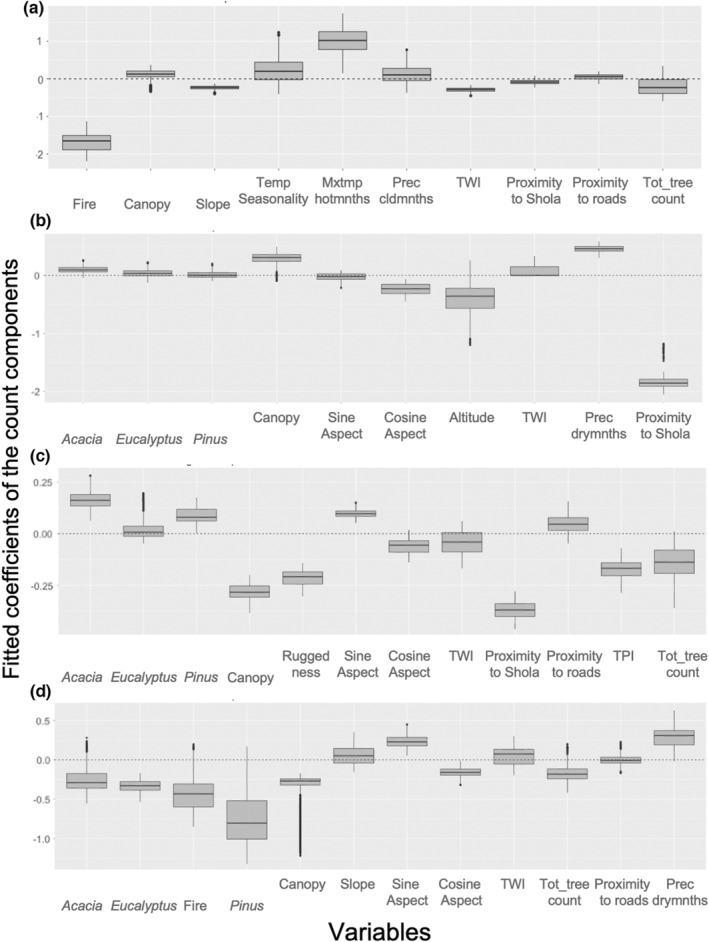
(a) Fitted coefficients of count components for *Lantana* sp., (b) Coefficients for *Cestrum* sp., (c) coefficients for Ageratina complex, and (d) coefficients for *Pteridium* sp. With the importance value (Table S3), we plotted the signal consistency of each variable: a box plot above the zero line indicates a consistent positive effect of the variable in the taxon regeneration; a box plot below the zero line indicates a consistent negative effect; and if box plot contains zero, the signal was not consistent in the fitted models.

*Lantana* was present in 41 plots with *Eucalyptus* canopy. Fire incidence and maximum temperature of the warm quarter were most important (VIV = 82.8%, 78.4%, respectively); higher fire incidence was negatively correlated with the *Lantana* occurrence, whereas higher dry quarter temperature was positively correlated with it. Among the zero‐inflation components, the canopy (VIV = 99.9%) and road networks (VIV = 97.9%) were important. The effect signals were consistent for canopy cover, suggesting a negative association of *Lantana* presence to canopy cover. The signals were inconsistent for the variable road networks (Figure 4).

*Cestrum* was absent in our Palani–Anamalai hills plots; hence, this analysis is limited to the plots of the Nilgiri hills. *Cestrum* was present in 45 plots of the 79 plots used in analyses. Precipitation in the dry quarter, TWI, and the area of Shola Forest within a hectare around the study plots were important for the invasion of *Cestrum* (VIV = 88.1%, 87.8%, and 95.7%, respectively). Higher rainfall in drier months and greater soil moisture were positively associated with *Cestrum* occurrence, whereas proximity to the Shola Forest is negatively associated with it (Figure 4). *Cestrum* presence showed consistently negative effect signals for canopy cover (VIV for zero‐inflation components 88.2%).

*Pteridium* was present in 92 plots. The basal area of *Pinus* trees and fire are important determinants of fern invasion (count and zero‐inflation components, respectively). *Pteridium* shows lower occurrence in plots with *Pinus* presence, although the signal of the effects is inconsistent. Fire incidences showed inconsistent signal effects for *Pteridium* presence.

*Ageratum conyzoides*, *Ageratum houstonianum*, and *Ageratina adenophora* occurred in 83 of 232 plots. The basal area of *Acacia*, canopy cover, proximity to Shola Forest, and TRI were important for the spread of the *Ageratina* complex (79.3%, 99.5%, 99.9%, and 84.0%, respectively). Plots with *Acacia* overstory had a higher probability of occurrence with regard to the *Ageratina* complex, and plots with greater canopy cover, proximity to Shola forests, and higher ruggedness had a lower probability of occurrence (Figure 4).

## 4 DISCUSSION

Our study examines the patterns of understory invasion in the timber stands, some of which are invasive overstory themselves. We report a region‐wide pattern of sequential phase invasion. Some of these patterns have important implications for ecological restoration actions currently underway in these habitats. All major timber overstory species were associated with some understory invasive species—*Eucalyptus* with *Lantana*, *Acacia* stands with the *Ageratina* complex, and *Pinus* with *Pteridium*.

### 4.1 Role of eucalyptus

In our study, *Eucalyptus* stands show high species richness of other invasive species in their understory. This is a similar pattern to China, where understory invasion in the *Eucalyptus* stands occurs significantly higher than in forests (Jin et al., 2015). In the Western Ghats, the spread of *Lantana* is a major threat (Joshi et al., 2015). Although *Lantana* has been known to invade lower elevations across a large part of India (Mungi et al., 2020), this study indicates the spread of *Lantana* to higher elevations, perhaps mediated by the presence of *Eucalyptus* akin to O'Loughlin and Green (2018). Apart from our study plots, we noticed *Lantana* in some of the higher reaches of the Western Ghats (~1840 m ASL), although in very small patches indicating an emerging problem that *Eucalyptus* stands may be facilitating.

### 4.2 Cestrum and other invasives

In our study, *Cestrum* was found only in the Nilgiri mountains where the extent of invasion is alarming, ~50% of all plots. It is widespread between 1800 and 2350 m elevation (Das, 2015). Although the information is available indicating such invasion in the Nilgiris (Das, 2015; Mohandass & Davidar, 2009; Suresh et al., 2017), a comparison of various montane areas was not conducted until now. Our findings indicate that unlike in the Nilgiris, *Cestrum* is not widespread in the Palani Hills, indicating regional differences in the spread of this invasive that requires further investigation. Canopy cover was negatively associated with the occurrence of both species—Lantana and *Cestrum—*and the *Ageratina* complex.

### 4.3 Implications for restoration

Shola Sky Islands have a large area (~218 sq. km) covered by invasive trees (Arasumani et al., 2019). Most of these areas are where planted trees have escaped the managed plantations and invaded neighboring Shola grasslands (Arasumani et al., 2019). Our data indicate that these timber stands appear to be hotbeds of invasion by other woody and herbaceous plants. The identity of these invasive species and patterns of invasion varies with the overstory species in the timber stands.

Most of our plots with *Eucalyptus* as the canopy species fall in the Western parts of the Shola Sky Islands system, which lies administratively in Kerala. Apart from a different management system, these plots also have a heavier rainfall regime (Karger et al., 2017). Relative rainfall can have a positive association with *Lantana* regeneration density (Debuse & Lewis, 2014). Increased light can result in the increased cover of *Lantana* in rainforests (Totland et al., 2005), but *Eucalyptus* stands are relatively open compared to native forests. With low variability in the light intensity gradient, the *Lantana* regeneration response may not be predominantly variable (Debuse & Lewis, 2014). In *Pinus* stands, leaf litter may hinder germination, hence the colonization of understory species (Senbeta & Teketay, 2001). In older stands, the litter thickness is high, and decomposition of such litter reduces with low temperature (Senbeta et al., 2002), possibly explaining the relative lack of invasive species in the *Pinus* stand understory. Finally, the stands of *Acacia mearnsii*, which are reported to show allelopathy, have a greater degree of shade and lack of humidity in the upper soil (Tassin et al., 2009). Our study follows the patterns mentioned above very closely.

At present, large areas are being planned for active restoration—removal of invasive trees—in both Kerala and Tamil Nadu (Correspondent, 2015). However, most action is targeted at *Acacia* and marginally at *Pinus*. Here, we present data showing that *Eucalyptus* stands should also be targeted for restoration when such activities are planned.

*Eucalyptus* species have been popular in compensatory afforestation programs across India (Vohra, 2021), Studies and reports abound in favor (Agarwal & Saxena, 2017; Pandit, 2018) and against the planting of *Eucalyptus* (Sikka et al., 2003). Our data suggest that, at least in some areas, these timber stands can facilitate secondary invasion and should thus not be permitted or actively managed to prevent invasion in the understory.

We do note that our study was conducted only above 1400 m elevation in the Western Ghats, and these patterns may be different elsewhere. We also note that this study does not present an exhaustive survey of invasives (see Table S2 for details) or invasion patterns in the region but rather investigates specific ecological contexts of understory invasives in an overstory of timber stands.

However, this study does indicate that large areas of the montane Shola Sky Islands that have been converted to timber stands are being impacted by the understory invasion of several species in ways that are specific to the habitat context and overstorey composition of these timber stands.

## AUTHOR CONTRIBUTIONS

**Varughese Jobin:** Conceptualization (lead); data curation (lead); formal analysis (lead); methodology (equal); project administration (supporting); visualization (equal); writing – original draft (lead); writing – review and editing (equal). **Arundhati Das:** Conceptualization (lead); formal analysis (supporting); methodology (equal); supervision (equal); validation (equal); visualization (equal); writing – original draft (supporting); writing – review and editing (equal). **C. P. Harikrishnan:** Conceptualization (supporting); data curation (supporting); methodology (supporting); writing – original draft (supporting). **Ritobroto Chanda:** Conceptualization (supporting); data curation (supporting); methodology (supporting); writing – original draft (supporting). **Swapna Lawrence:** Conceptualization (supporting); data curation (supporting); methodology (supporting); writing – original draft (supporting). **V. V. Robin:** Conceptualization (supporting); formal analysis (supporting); funding acquisition (lead); methodology (equal); project administration (lead); resources (lead); supervision (equal); validation (equal); visualization (equal); writing – original draft (supporting); writing – review and editing (equal).

## CONFLICT OF INTEREST STATEMENT

The authors declare no competing interests.

## Supporting information


Tables S1–S3

## Data Availability

We provide the data file for our study, and the DOI number for the Dryad repository is https://doi.org/10.5061/dryad.3bk3j9kpc.
