# Supplementary material for: Patterns of understory invasion in invasive timber stands of a tropical sky island
Source: Ecol Evol. 2023 Apr 13;13(4):e9995. doi: 10.1002/ece3.9995 (PMC10099487; doi:10.1002/ece3.9995)
Supplement: Supplementary file 1 — Tables S1–S3 [file ECE3-13-e9995-s001.docx]

**Supplementary**

**Table 1: List of putative explanatory variables of Invasive regeneration and their categorisation.**

| **Model Component** | **Type of Variable** | ***Cestrum aurantiacum*** | ***Lantana camara*** | ***Pteridium aquilinum*** | ***Ageratina Complex*** |
| --- | --- | --- | --- | --- | --- |
| **Count components** | **Compositional factors** | Basal area of *Acacia* |  | Basal area of *Acacia* | Basal area of *Acacia* |
|  |  | Basal area of *Eucalyptus* | Basal area of *Eucalyptus* | Basal area of *Eucalyptus* | Basal area of *Eucalyptus* |
|  |  | Basal area of *Pinus* |  | Basal area of *Pinus* | *Pinus* (Basal area) |
|  | **Structural factors** | Canopy cover | Canopy cover | Canopy cover | Canopy |
|  |  |  | Total count of trees | Total count of trees | Total count of trees |
|  | **Topographic factors** | Sine aspect | Sine aspect | Sine aspect | Sine aspect |
|  |  | Cosine aspect | Cosine aspect | Cosine aspect | Cosine aspect |
|  |  | Ln(elevation) |  |  |  |
|  |  |  | Slope | Slope |  |
|  |  | TWI | TWI | TWI | TWI |
|  |  |  |  |  | TRI |
|  |  |  |  |  | TPI |
|  | **Climatic factors** |  | Temp Seasonality |  |  |
|  |  |  | Precipitation in cold quarter |  |  |
|  |  |  | Maximum temperature in hot quarter |  |  |
|  |  |  |  | Min temperature in cold quarter |  |
|  |  | Precipitation in dry quarter |  | Precipitation in dry quarter |  |
|  | **Landscape factors** | ln_shlbfr5ha | ln_shlbfr5ha |  | ln_shlbfr5ha |
|  |  |  | rds_lngth_5ha | rds_lngth_5ha | rds_lngth_5ha |
|  | **Others** |  | Fire | Fire |  |
| **Zero-inflation component** |  | Canopy | Canopy |  | Canopy |
|  |  | TWI | TWI |  | TWI |
|  |  |  | rds_lngth_5ha | rds_lngth_5ha | rds_lngth_5ha |
|  |  |  |  | Fire |  |
| **Random variables** |  | Site | Type of timber stand | Type of timber stand | Type of timber stand |
| **References for selection of variables** |  | (Ojunga et al. 2020; Junaedi 2013) | (Sundaram and Hiremath 2012; Prasad 2012); | (Dolling 1999; Amouzgar et al. 2020; de Silva and Matos 2006) | (Lamsal et al. 2019; F. Wan et al. 2010; Yuan and Wen 2018; Parthasarathy and Pragasan 2011) |

**Table 2: Association value between stand types and colonising invasive species, with their statistical significance values. (Association value>0.4, usually diagnostic for the target vegetation unit)**

| **Colonising species** | **Association value** | **Statistical significance** |
| --- | --- | --- |
| ***Eucalyptus* stands** | | |
| *Lantana camara* | 0.486 | 0.001 |
| *Eucalyptus spp.* | 0.459 | 0.001 |
| *Urena lobata* | 0.198 | 0.080 |
| *Ageratum conyzoides* | 0.191 | 0.094 |
| *Solanum sp.* | 0.174 | 0.174 |
| *Ageratum houstonianum* | 0.148 | 0.321 |
| *Ipomea purpurea* | 0.116 | 0.594 |
| *Achyranthes aspera* | 0.111 | 0.611 |
| Malvaceae | 0.092 | 1.000 |
| Meliaceae | 0.092 | 1.000 |
| *Clitoria ternetea* | 0.092 | 1.000 |
| *Desmodium uncinatum* | 0.092 | 1.000 |
| *Asparagus racemosus* | 0.092 | 1.000 |
| *Oxalis corniculata* | 0.092 | 1.000 |
| Asteraceae | 0.092 | 1.000 |
| Apiaceae | 0.092 | 1.000 |
| *Tridax procumbens* | 0.092 | 1.000 |
| Urticaceae | 0.092 | 1.000 |
| ***Pinus* stands** | | |
| *Solanum mauritianum* | 0.131 | 0.52 |
| *Pinus radiata* | 0.124 | 0.643 |
| ***Acacia*+*Eucalyptus* stands & mixed stands** | | |
| *Acacia mearnsii* | 0.378 | 0.001 |
| ***Eucalyptus* & *Pinus* Stands** | | |
| *Pteridium aquilinum* | 0.205 | 0.061 |
| ***Acacia* & *Acacia*+*Eucalyptus* & *Eucalyptus* stands** | | |
| *Ageratina adenophora* | 0.333 | 0.002 |
| ***Acacia* & *Acacia*+*Eucalyptus* & *Pinus* stands** | | |
| *Cestrum aurantiacum* | 0.263 | 0.015 |

**Table 3: List of Variable importance Values for Invasive regeneration**

| **Variable Importance Value** | | | | | |
| --- | --- | --- | --- | --- | --- |
|  | **Variables** | ***Lantana*** | ***Cestrum*** | ***Ageratina* complex** | ***Pteridium*** |
| **Count component** | *Acacia* | NA | 28.0 | ***79.3*** | 44.9 |
|  | *Eucalyptus* | NA | 22.4 | 26.5 | 68.1 |
|  | Fire | ***100.00*** | NA | NA | 63.1 |
|  | *Pinus* | NA | 22.4 | 52.1 | ***75.7*** |
|  | Canopy cover | 28.16 | 41.7 | ***99.5*** | 67.7 |
|  | Slope | 39.46 | NA | NA | 29.6 |
|  | Ln_elevation | NA | 29.4 | NA | NA |
|  | Sine.aspect | NA | 21.9 | 44.5 | 53.3 |
|  | Cosine.aspect | NA | 36.9 | 28.7 | 40.8 |
|  | Temp.seasonality | 35.00 | NA | NA | NA |
|  | Mxtmp_hotmnths | ***72.23*** | NA | NA | NA |
|  | Prec_cldmnths | 28.49 | NA | NA | NA |
|  | Prec_drymnths | NA | ***88.1*** | NA | 41.0 |
|  | TWI | 47.14 | ***87.8*** | 27.5 | 29.0 |
|  | Tot_ct_tree | 25.68 | NA | 33.1 | 32.0 |
|  | ln_shlbfr5ha | 25.14 | ***95.7*** | ***99.9*** | NA |
|  | rds_lngth_5ha | 28.89 | NA | 36.2 | 25.7 |
|  | TRI | NA | NA | ***84.0*** | NA |
|  | TPI | NA | NA | 45.9 | NA |
|  | | | | | |
| **Zero-inflation component** | Canopy cover | ***99.95*** | ***88.2*** | 41.8 | NA |
|  | TWI | 51.31 | NA | 40.2 | NA |
|  | rds_lngth_5ha | ***98.39*** | NA | 51.4 | 37.2 |
|  | Fire | NA | NA | NA | ***86.4*** |
|  | ln_shlbfr5ha | NA | 32.8 | NA | NA |
